# Relating individual cell division events to single-cell ERK and Akt activity time courses

**DOI:** 10.1038/s41598-022-23071-6

**Published:** 2022-10-27

**Authors:** Alan D. Stern, Gregory R. Smith, Luis C. Santos, Deepraj Sarmah, Xiang Zhang, Xiaoming Lu, Federico Iuricich, Gaurav Pandey, Ravi Iyengar, Marc R. Birtwistle

**Affiliations:** 1 Department of Pharmacological Sciences, Icahn School of Medicine at Mount Sinai, New York, NY USA; 2 Department of Neurology, Center for Advanced Research on Diagnostic Assays, Icahn School of Medicine at Mount Sinai, New York, NY USA; 3 Department of Chemical and Biomolecular Engineering, Clemson University, Clemson, SC USA; 4 School of Computing, Clemson University, Clemson, SC USA; 5 Department of Genetics and Genomic Sciences, Icahn School of Medicine at Mount Sinai, New York, NY USA

**Keywords:** Cell division, Cell signalling, Cellular imaging

## Abstract

Biochemical correlates of stochastic single-cell fates have been elusive, even for the well-studied mammalian cell cycle. We monitored single-cell dynamics of the ERK and Akt pathways, critical cell cycle progression hubs and anti-cancer drug targets, and paired them to division events in the same single cells using the non-transformed MCF10A epithelial line. Following growth factor treatment, in cells that divide both ERK and Akt activities are significantly higher within the S-G2 time window (~ 8.5–40 h). Such differences were much smaller in the pre-S-phase, restriction point window which is traditionally associated with ERK and Akt activity dependence, suggesting unappreciated roles for ERK and Akt in S through G2. Simple metrics of central tendency in this time window are associated with subsequent cell division fates. ERK activity was more strongly associated with division fates than Akt activity, suggesting Akt activity dynamics may contribute less to the decision driving cell division in this context. We also find that ERK and Akt activities are less correlated with each other in cells that divide. Network reconstruction experiments demonstrated that this correlation behavior was likely not due to crosstalk, as ERK and Akt do not interact in this context, in contrast to other transformed cell types. Overall, our findings support roles for ERK and Akt activity throughout the cell cycle as opposed to just before the restriction point, and suggest ERK activity dynamics may be more important than Akt activity dynamics for driving cell division in this non-transformed context.

## Introduction

The mammalian cell cycle is in large part driven by growth factor activation of the Ras-ERK^1–5^ and the PI3K-Akt^2,6–11^ pathways. Growth factors cause auto-phosphorylation of receptor tyrosine kinases (RTKs). For the ERK pathway, RTK phosphorylation recruits the guanine exchange factor SOS to the membrane, catalyzing the exchange of GDP for GTP bound to Ras, initiating Raf activation^4–6,12–14^. This in turn activates the MEK-ERK phosphorylation cascade. When activated, the effector kinases ERK1/2 translocate from the cytoplasm to the nucleus and activate transcriptional regulators such as Elk-1 and CREB^15–17^. These transcriptional regulators induce immediate early genes (IEGs) like *c-fos*^15,16^ that then contribute to the expression of cyclin D1^4,6,8,17–19^, a key step in S-phase entry^20^.

RTK activation can also initiate Akt pathway signaling. RTK autophosphorylation recruits adapter proteins like insulin receptor substrate (IRS-1) and GRB2-associated binder (GAB)^2,21–23^. These proteins in turn recruit Phosphatidylinositol (PtdIns) 3-kinase (PI3K) to the membrane^2,19,24,25^ where it phosphorylates the membrane phospholipid PtdIns (4,5) P2 (PIP2), generating PtdIns (3,4,5) P3 (PIP3). PIP3 recruits pleckstrin homology domain (PH)-containing proteins to the membrane such as phosphatidylinositol-dependent kinase-1 (PDK1)^26^ and the serine/threonine protein kinases Akt1/2^19,26^. PDK1 phosphorylates Akt’s activation loop followed by mTORC2 phosphorylation of a second site on Akt for full activation^6,7,19^. This doubly phosphorylated, activated Akt promotes cell cycle progression by: (1) promoting protein translation via 4E-BP and p70S6K^6,19^, (2) promoting cyclin D1^7,18^ CDK4/6, c-Myc, and E2F activity^27^ and (3) inhibiting p21 and p27^28^ (cyclin-dependent kinase inhibitors).

While ERK and Akt pathways have established roles prior to the restriction point marked by S-phase entry, the extent to which they are informative of cell cycle completion after S-phase is less clear. Beyond S-phase, studies suggest that Ras-ERK^29–36^ and PI3K-Akt^26,37,38^ may contribute towards regulating G2 progression. ERK activity was shown to play a role in the duration of DNA damage-induced G2 arrest^39^. Transient ERK activity maintains G2 arrest, whereas sustained ERK activity promotes escape by reducing p53 levels, and inducing the expression of pro-mitotic factors such as Plk1 and cyclin B^36^. Akt activity also contributes to G2-M progression as its inhibition is associated with reduced cyclin B levels, promoting Chk1 activity and G2 arrest^37^. These observations motivate a closer look at determining how ERK and Akt dynamics are informative of cell cycle completion after the canonical restriction point.

On a single cell level, both ERK and Akt activity dynamics have substantial cell-to-cell and dynamic variation, exhibiting complex pulses and more simple steady activity^1,24,40–48^. Such variation, when coupled with the observations that cell cycle progression is also heterogeneous^1,49,50^, have prompted investigations into the correlation between dynamics and cell cycle fate in single cells. What determines proliferation on a single cell level? What relative contributions do ERK and Akt activity have to the decision of individual cells to divide? Much prior work has focused on activity dynamics. Both Ras-ERK^51–53^ and PI3K-Akt^54^ exhibit biphasic growth factor-induced activation dynamics, with a transient peak followed by sustained activity hours later. The dynamics of each phase contributes differently towards driving progression to S-phase and is cell type dependent^54–56^. Live-cell imaging and analysis of recently divided sister cells reveal that time-integrated ERK activity has some predictive power of the timing to S-phase entry^1^. Time-integrated ERK dynamics were found to influence proliferation decisions in daughter cells^45^. Predicting PC12 cell differentiation/proliferation decisions required both ERK and Akt activity dynamics to best define the decision boundary between these two cell fate outcomes^57^. Yet, the extent to which both ERK and Akt activities throughout the cell cycle are associated with division in single cells remains unclear.

Here, we use live-cell imaging to pair measurements of growth factor-induced ERK and Akt activity to cell division outcomes in the same single cells. We aim to assess the extent to which these activities are associated with cell cycle progression beyond S-phase entry to single cell division responses, using the well-established non-transformed breast epithelial MCF10A cell line, a model system that is commonly used to study epithelial signaling biology and cell division control^2,58–63^. We found that following treatment of synchronized cells with growth factors EGF and insulin, both ERK and Akt activity are significantly higher within the S-G2 interval in dividing cells. Such differences were much smaller in the pre-S-phase window, which is traditionally associated with ERK and Akt activity dependence^54–56^, suggesting unappreciated roles for ERK and Akt in S through G2. ERK activity was more strongly associated with cell division events, suggesting Akt activity may play a smaller role in this context. Interestingly, we found that ERK and Akt activities are less correlated in cells that divide. Network reconstruction experiments demonstrated that this correlation behavior was not due to crosstalk, as ERK and Akt do not interact in this context, in contrast to other cell types^64^. Overall, our findings support roles for ERK and Akt activity throughout the cell cycle as opposed to just before the restriction point, and suggest ERK activity dynamics may be more important than Akt activity dynamics for driving cell division in this non-transformed context.

## Results

### Association of cell division events with univariate ERK and Akt dynamics

**Figure 1:**
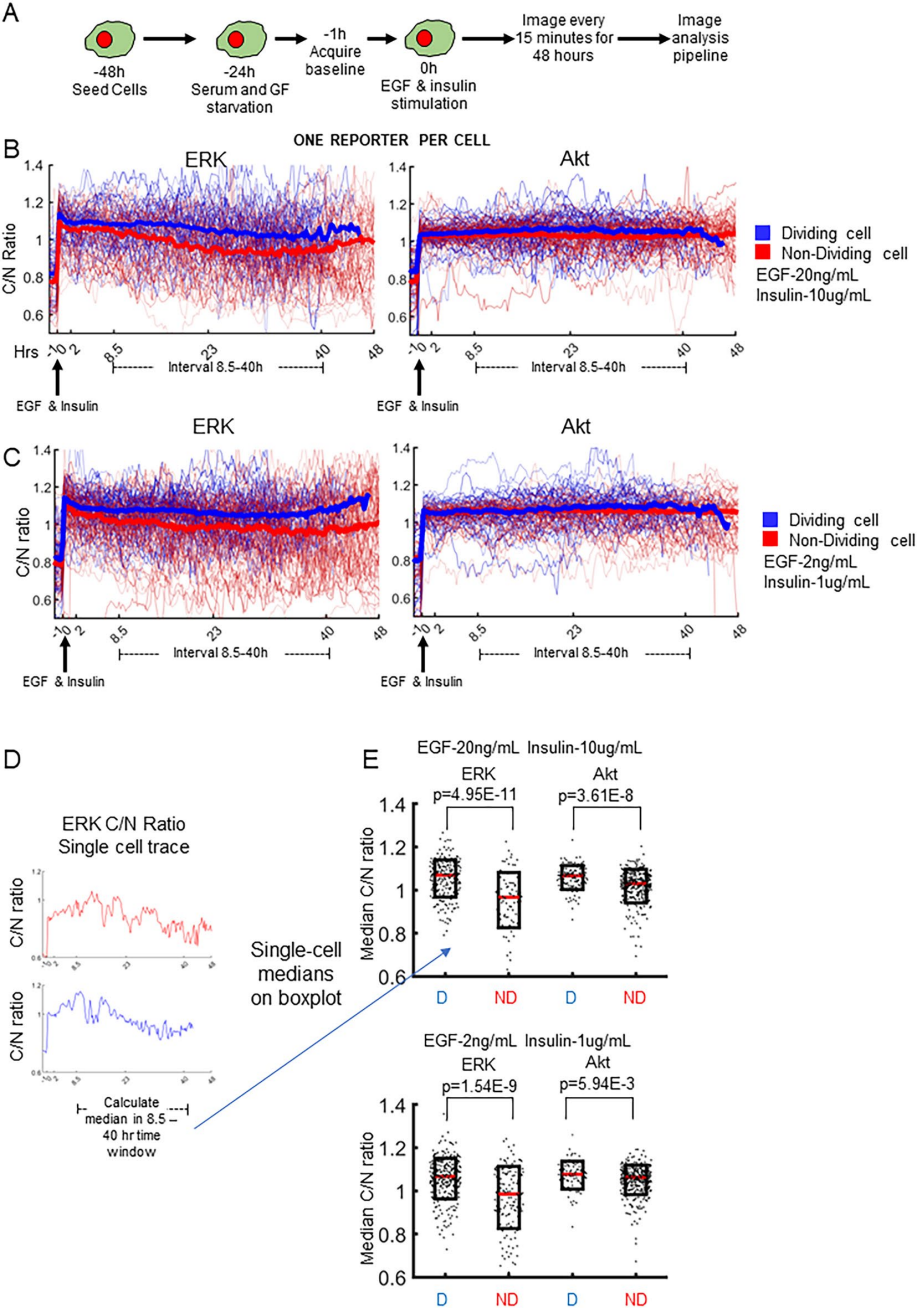
Evaluating the association of cell division with univariate single cell ERK or Akt dynamics. In these experiments, cells were either expressing the ERK or the Akt KTR. (**A**) Cell treatment workflow for pairing single cell KTR dynamics to cell division. ERK or Akt KTR expressing MCF10A cells were seeded, allowed to attach overnight, and then serum and growth factor starved. Following starvation, baseline images were acquired, cells were treated with EGF and insulin, and then imaged every 15 min for 48 h. Images were quantified using the analysis pipeline described in the “Methods”. (**B**, **C**) Quantified ERK or Akt KTR dynamics paired to division events for EGF and Insulin doses that match those used in culture medium (**B**) or tenfold less (**C**). Single cell traces of dividing (blue) and non-dividing (red) cells are shown with thin lines, and population median (per time point) is shown with thick lines. (**D**) Left, representative single cell trace of ERK KTR for a dividing (blue) or non-dividing (red) cell. Median ERK activity within the 8.5–40 h interval for each cell becomes a single dot in the boxplots. (**E**) notBoxplots for single cell median ERK or Akt activity within the 8.5–40 h interval for EGF and Insulin doses that match those used in culture medium (top) or tenfold less (bottom). p-values for the right-tailed rank-sum test were calculated at the 95% confidence interval. D, dividing; ND, non-dividing.

To evaluate associations between ERK or Akt signaling dynamics and cell division, we first conducted a series of live cell imaging experiments in MCF10A cells that express either ERK^41,65^ or Akt^43^ kinase translocation reporters (KTRs), but not yet both at once, and paired those single cell dynamics to cell division events (Fig. 1A). We first verified that cell cycle progression and division are related to ERK and Akt activity dynamics in MCF10A cells using small molecule inhibitor experiments (Fig. S1). KTR-expressing cells were G0-synchronized by serum and growth factor starvation for 24 h. After acquiring 1 h of baseline ERK or Akt activity, cells were treated with EGF and insulin, growth factors that promote cell division in MCF10A cells^66^. Images were acquired every 15 min for 48 h, and then single-cell data for kinase activity and division outcome were extracted using custom-built image processing pipelines (see “Methods”). Dynamic regimes of KTR specificity were determined using two independent (four total) MEK and Akt inhibitors (Fig. S2). ERK KTR was found to be specific in all regimes explored here, whereas the Akt KTR was found to be specific >  ~ 1 h after EGF and insulin co-stimulation. Variability from cell-to-cell at a particular moment in time, and across a single cell over time, suggested that the probes had informative dynamic range outside of the acute time period following the ~ 4–8 h post-growth factor stimulation (Supplementary Figs. S9, S10).

Single cell traces of ERK or Akt activity (thin lines) along with the population median (bold line) show rapid activation following growth factor treatment, which largely persists for the duration of the experiment (Fig. 1B, C). In (blue) dividing cells, population median ERK and Akt activity dynamics are higher throughout the cell cycle compared to non-dividing cells, with larger differences evident for ERK. In the pre-S-phase entry window (~ < 8 h after growth factor treatment), there are slight differences between dividing and non-dividing cells in terms of population median ERK and Akt dynamics. These differences grow larger in the subsequent 8.5–40 h interval post growth factor addition, which largely corresponds to S and G2 phases. These trends were also evident with ten-fold less concentration of growth factors (Fig. 1C). These results suggest that ERK and Akt activity may have importance after S through G2 phase.

To assess the statistical significance of this finding, we calculated the median ERK or Akt activity for individual single cells within the 8.5–40 h window post-growth factor treatment, and then compared median activity between dividing and non-dividing cells with the single tailed rank-sum test (Fig. 1D, E). Individual dots in the boxplot represent the median ERK or Akt activity calculated within the 8.5–40 h interval in a single cell. These median single cell activities were significantly different in dividing vs. non-dividing populations (Fig. 1E). ERK activity was more significantly different in dividing vs non-dividing cells than Akt activity. Nevertheless, there is substantial overlap in the two populations, indicating other factors influencing cell division events. We also cannot rule out that other dynamic features in addition to the 8.5–40 h median may be additionally informative.

### Measurements of both ERK and Akt dynamics in the same single cells correlated with cell division fate

**Figure 2:**
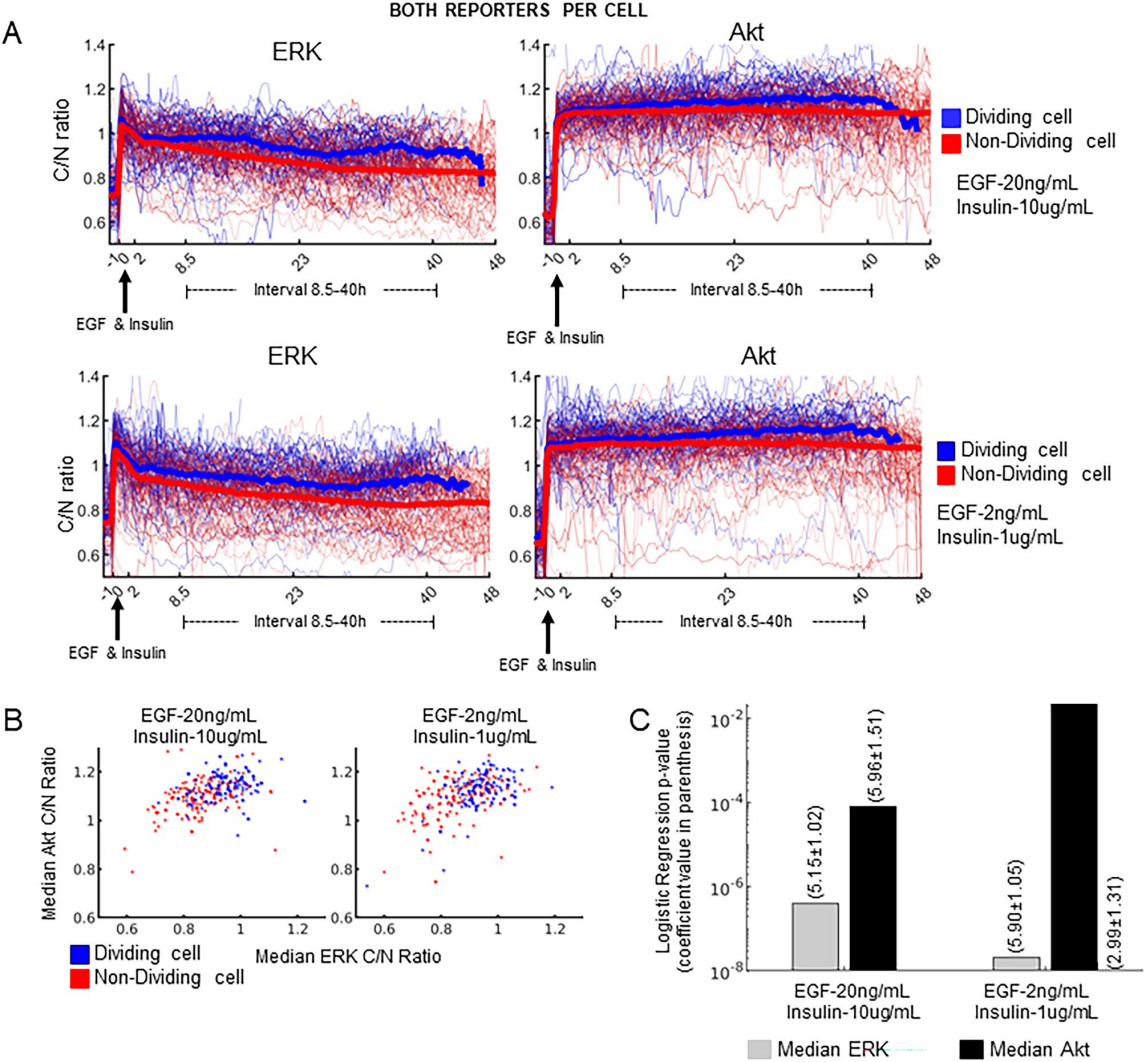
Evaluating the association of cell division with paired, bivariate single-cell ERK and Akt dynamics. In these experiments, cells were expressing both the ERK and Akt KTR simultaneously. (**A**) Quantified ERK and Akt dynamics for EGF and Insulin doses that match those used in culture medium (top) or ten-fold less (bottom). Single cell traces of dividing (blue) and non-dividing (red) cells are shown with thin lines, and population median (per time point) is shown with thick lines. (**B**) Scatter plot of ERK vs. Akt KTR median activity in the 8.5–40 h window from 150 randomly sampled cells. Each dot is a single cell. Dividing cells are blue and non-dividing cells are red. Left and right are high and low growth factor concentrations, respectively. (**C**) Statistical significance of logistic regression coefficients for either ERK or Akt median activity (8.5–40 h), with regression coefficients shown in parentheses above the respective bars (uncertainty is standard error).

As shown above, ERK and Akt activity dynamics alone contain information about subsequent cell division. Do ERK and Akt activity covariations also correlate with cell division fate? Is one more strongly associated than the other? To answer these questions, we performed a similar experiment as described above using dual reporter expressing MCF10A cells (see “Methods”). For the duration of the time course, population median ERK and Akt dynamics are again elevated in dividing cells compared to non-dividing cells (Fig. 2A), with larger differences observed in the 8.5–40 h interval. Median ERK and Akt dynamics together had some ability to stratify dividing cells, but again, there is substantial overlap (Fig. 2B). Logistic regression indicated that median ERK activity was more strongly associated with cell division events as compared to median Akt activity (Fig. 2C). These results were confirmed in an independent experiment (Fig. S3). Moreover, imaging data acquired at a 5-min interval corroborated these findings (Fig. S8). We conclude that Akt dynamics add comparatively little new information to ERK dynamics for predicting single cell division events in this context. Although the median in the 8.5–40 h post-growth factor window was analyzed here, we cannot rule out the importance of other dynamic features such as pulsing, although in our datasets we did not observe significant pulsing^1,24,40–48^ (perhaps due to experimental differences such as subconfluency and serum-starvation—see “Discussion”). Moreover, because of strong growth factor stimulation and potential probe saturation in the 0–8.5 h window, we cannot rule out the importance of this earlier signaling window either.

### Inferring the topology of the ERK-Akt network

**Figure 3:**
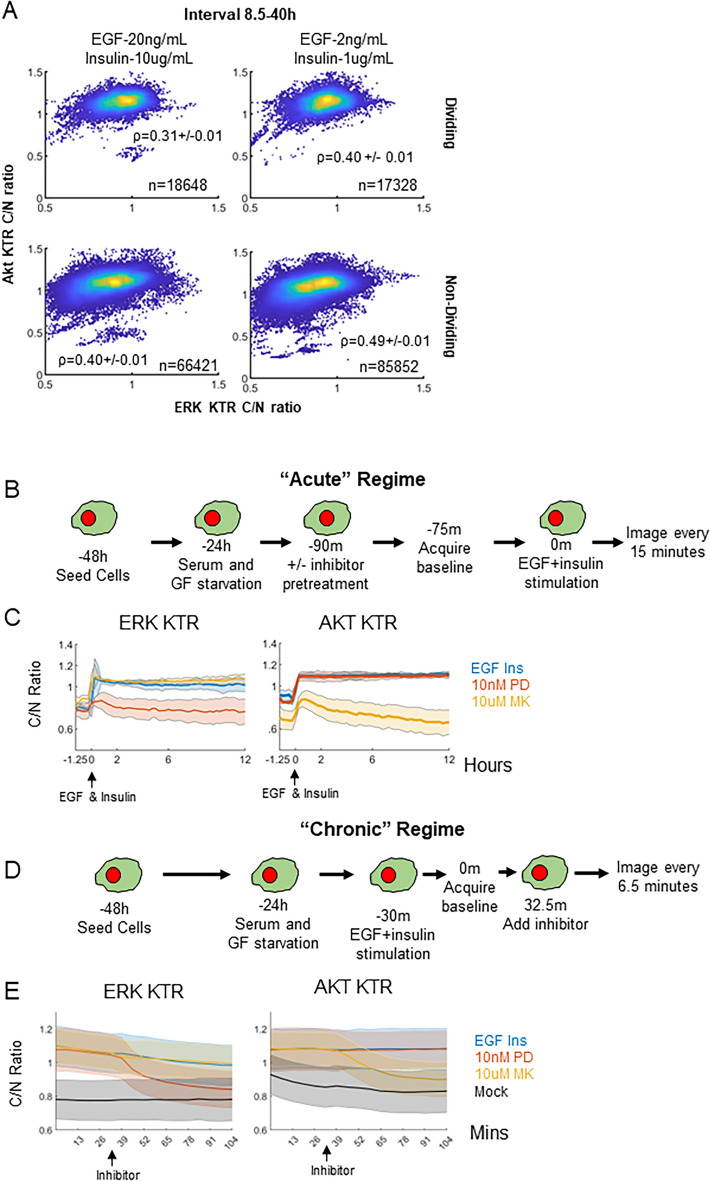
Investigating properties of the ERK and Akt network. (**A**) Single cell ERK and Akt activity plotted across all time points within the 8.5–40 h interval for dividing and non-dividing cells. These cells expressed both the ERK and Akt KTR. Pearson correlation coefficient, along with the number of cell-time datapoint combinations are indicated. Uncertainty in the correlation coefficients is calculated as described in “Methods”. (**B**) Cell treatment workflow for network reconstruction in the “acute” regime. Single ERK or Akt KTR expressing MCF10A cells were seeded, allowed to attach overnight, and then serum and growth factor starved. Following starvation, inhibitor was added (PD: PD0325091; MK: MK2206), baseline images were acquired, cells were treated with EGF and insulin, and then imaged every 15 min for 12 h. Images were quantified using the analysis pipeline described in the “Methods”. (**C**) Quantified ERK and Akt activity dynamics in the acute regime. Solid lines are population median (per time point), and shaded areas denote the standard deviation across cells. (**D**) Cell treatment workflow for network reconstruction in the “chronic” regime. Dual ERK and Akt KTR expressing MCF10A cells were seeded, allowed to attach overnight, and then serum and growth factor starved. Following starvation, EGF and insulin were added, baseline images were acquired, cells were treated with inhibitors, and then imaged every 6.5 min for the remaining ~ hour. Images were quantified using the analysis pipeline described in the “Methods”. (**E**) Quantified ERK and Akt activity dynamics in the chronic regime. Solid lines are population median (per time point), and shaded areas denote the standard deviation.

Information content is related to correlation, so we investigated the extent to which ERK and Akt dynamics in the same single cells were correlated, looking across every cell and every time point (Fig. 3A, replicate Fig. S3D). Interestingly, in dividing cells, single cell ERK and Akt dynamics within the 8.5–40 h window are significantly less correlated than in non-dividing cells, at both high and low growth factor doses. Network topology can strongly influence correlated behaviors. In different studies, ERK and Akt have been reported to exhibit very different network behavior, such as cross-pathway activation, inhibition^6,18,19,23,64,67–71^ and non-interaction^21,64,72^. Factors such as cell type and growth factor context can influence these discrepant network topologies^3,64^. Previous work conducted in panel of growth factors and cell lines show varying probabilities of forming an interaction network edge between ERK and Akt^64^. The differences in network edge formation can affect downstream signaling and cell fate decisions^3^. Could ERK and Akt network topology give insight into the division-related correlated behaviors observed above?

To reconstruct the ERK and Akt network in MCF10A cells, we implemented recent theory from our lab that specifies a sufficient experimental design for such tasks, based on perturbation time course data^73^. Specifically, for this 2-node network, three time course experiments should be done: response of ERK and Akt activity to EGF and Insulin co-treatment with (1) no inhibitor; (2) an ERK pathway inhibitor; and (3) an Akt pathway inhibitor. Additionally, we wanted to understand whether the network would be different in the acute phase of growth factor treatment from a serum starved state vs. the “chronic” condition where ERK and Akt activities are steady over time, particularly because these time regimes seem to have different biological information encoded for cell division decisions.

In the acute regime (Fig. 3B), MCF10A cells expressing either ERK or Akt KTR were seeded, serum and growth factor starved for 24 h, and then pretreated with either a MEK (PD0325901) or an Akt (MK2206) inhibitor for 30 min. The concentrations utilized were determined via titration experiments to ensure minimum possible doses were being used (Figs. S1, S2, S4). Following drug treatment, baseline KTR activity was acquired every 15 min for 1 h. Then, we treated cells with EGF and insulin and imaged. Single cell traces (thin) and population median activity (bold) were calculated for each condition, showing robust ERK and Akt activation (Fig. 3C). MEK inhibition ablates ERK activation and has a negligible effect on Akt activation (Fig. 3C). Akt inhibition ablates Akt activation and has a negligible effect on ERK activation (Fig. 3C). Although the Akt KTR may reflect kinase activity other than Akt in this acute stimulus regime, the fact that complete inhibition of the ERK pathway has negligible impact on the Akt KTR readout means that ERK does not impact Akt or the others. These results show that in the acute stimulus regime, ERK and Akt exhibit negligible crosstalk after treatment with EGF and insulin.

In the chronic regime, cells were pretreated with either EGF and insulin for 30 min followed by 30 min of baseline acquisition, leading to robust ERK and Akt activation (Fig. 3D, E, Replicates in S5). Akt inhibition reduces Akt activity, as expected, but negligibly affects ERK activity. MEK inhibition reduces ERK activity, as expected, but does not appreciably affect Akt activity. These conclusions are also consistent when a second set of MEK and Akt inhibitors are used (Fig. S4). Therefore, in the chronic regime ERK and Akt also do not exhibit appreciable cross pathway interactions after EGF and insulin co-treatment. We conclude it is unlikely that crosstalk interactions account for correlations that change in dividing vs. non-dividing cells.

## Discussion

Binary single-cell responses, like division, to perturbations such as growth factor and drug treatments, are almost universally heterogeneous even in clonally derived populations. However, biochemical features associated with heterogeneous fates, present either before the perturbation, or from dynamics following the perturbation, are not well established. The ability to predict such binary responses would not only reflect a deep and fundamental understanding of the systems governing important cellular responses, but also have significant translational applications such as antibiotic resistance, tissue engineering, and anticancer therapy, where the fates of single cells can be of great importance. Here, we investigated growth-factor induced cell division fates in the well-studied, non-transformed mammalian epithelial cell line MCF10A, and how they may be associated with the dynamics of two central signaling pathways, PI-3 K/Akt and Ras/ERK. Answering such questions requires single-cell, non-destructive analysis of biochemical features, in this case ERK and Akt activities, that are paired to the eventual cell division outcome. They also must be carried out in a high-throughput manner to observe enough events to make statistically-supported conclusions. After setting up this experimental system and understanding its ranges of validity, we learned that (1) ERK and Akt activities are higher in the 8.5–40 h window after growth factor treatment in cells that divide, suggesting underappreciated roles post-restriction point, into S and G2 phases; (2) median activities in this time window are associated with cell division outcome, and ERK activity was more strongly associated than Akt activity; (3) ERK and Akt activities are less correlated in cells that divide; and (4) ERK and Akt do not exhibit crosstalk in this system, so division-related correlation is unlikely related to crosstalk.

We have performed these studies in the MCF10A cell line, a well-established model for non-transformed epithelial cells. An obvious next question is how the relationships between ERK, Akt and cell division found here translate to different cell lines, and transformation contexts. Many other cell lines are cancer-derived and genetically unstable, and/or contain multiple alterations to the systems that control cell cycle progression and division. A firm understanding of how ERK and Akt systems control the cell cycle in a system such as MCF10A is an important foundation for subsequently understanding how it may be altered in other cell lines, and also across different microenvironmental contexts, such as confluent settings. Indeed, there is a growing body of work that focuses on answering fundamental cell biological questions using studies on the MCF10A system alone (e.g.,^74^). It is appealing to consider MCF10A as an emerging model system for mammalian epithelial cells.

Nearly all the cells we observed had relatively simple dynamics for ERK and Akt activity, a rise then a somewhat constant higher than baseline steady-state. Other recent single cell studies have reported pulsatile ERK dynamics^1,44,75,76^ using a 5 min acquisition frequency. When we used a 5 min acquisition frequency, we still did not observe pulsatile activity, although this increased frequency of acquisition required shorter exposure times which increased noise in the images. Pulsatile vs non-pulsatile dynamics may be related to differences in growth factor concentration, the reporters used, being FRET-based^77^ or translocation based^65^. No live-cell imaging probe is perfect and of course has its drawbacks, some of which may be related to off-target responses, which may partly explain our Akt activity data in the “acute” phase first following growth factor treatment. For example, kinases other than Akt may recognize and phosphorylate the FOXO1-based Akt KTR docking site^78–82^. EGF and insulin stimulation may also promote activation of such kinases including PLK1^83^, SGK and PKA^84^. Another aspect may have to do with cell–cell contact and density. In our study, cells were seeded at low density and serum/growth factor-starved prior to analysis, whereas pulsatile signaling was reported in high density environments in asynchronously cycling settings^1,76^. Yet others have found pulsatile dynamics can induce different sets of genes as compared to sustained dynamics^41^. However, phenotypic consequences, at least in terms of cell proliferation, still seem to be related to simple time-integrated signaling dynamics^1,45^, similar to what we found here.

ERK and Akt activity dynamics are only a subset of the potentially important variations that drive phenotypic variability in cell division responses. ERK activity was substantially more significant than Akt activity. This reinforces Akt activity as perhaps more relevant for cell maintenance and health, and more as a “checkpoint” for division but not a significant driver, at least in the studied system. As noted above, cell contacts and density are important. Such phenomena may potentially be controlled through micropatterning experiments, where cell shape and placement can be carefully controlled^85,86^. Cell “state”, corresponding to different epigenetic and/or metabolic states of cells prior to the experiment, has been reasonably well documented ubiquitously, and can contribute to variability, although is difficult to assess in the “track and follow” manner that can be done with live-cell kinase reporters. Metabolic or organelle abundance variability may also contribute^87,88^. Of course, there are other pathways and biochemical correlates that are likely important, such as a balance between p53 and p21 and/or CDK2 activity^49,62,89^. Given the multitude of fluorescent proteins, and improvements in cell tracking from non-labeled bright field images^90,91^, one may be able to measure more important biochemical readouts simultaneously for such purposes. There are also multiple checkpoints between growth factor treatment and cell division, such as the restriction point, and DNA damage checkpoints, that may contribute. Monitoring division with probes like the Fucci system that gives readouts of each cell cycle phase may help explore such phenomena^92^.

An interesting aspect of our study was differences between dividing and non-dividing cells in the time period that corresponds to S/G2 phases of the cell cycle. The roles of growth factor signaling through ERK and Akt pathways historically focused on passing the restriction point into S-phase^93^. Although we did not see large differences pre-S-phase, this may have been due to probe saturation in the acute period following growth factor stimulation. Nevertheless, our results suggest potential functional roles for ERK and Akt beyond this canonical understanding. Indeed, a recent study found time-integrated ERK activity in a mother cell’s G2 phase influenced the cell cycle progression in the subsequently daughter cells^45^. The mechanisms that may be driving such functional roles are a potentially interesting area of future study.

We also studied the ERK and Akt activity network, since we found that ERK and Akt activity are less correlated with each other in dividing cells compared to non-dividing cells. We found that the observed differences in correlation are likely not a result of network topology as ERK and Akt do not appreciably interact. This lack of interaction is surprising given that some prior studies describe these pathways as exhibiting cross pathway interactions^21,23,64^, albeit in other cell lines and in response to other growth factors. However, other studies in MCF10A cells across a panel of different growth factors show that ERK and Akt are insular, and do not interact^64^. Similar to MCF10A cells, 32D-EpoR; BaF3-EpoR; CFU-E cell lines exhibit minimal ERK and Akt cross pathway interaction under erythropoietin stimulation, a growth factor that activates both ERK and Akt^72^. These studies reveal that in non-interacting pathways, differences in protein expression influence the flow of erythropoietin signaling^72^. Therefore, in our model system, it is possible that the observed differences in ERK and Akt correlation may arise from differences in protein expression across dividing and non-dividing cells. It may also be that differences in upstream signaling capacity to ERK and Akt may be related. Characterizing the differences in protein expression level in single cells and following cell signaling and cell division can provide insight; but this becomes a quite challenging experiment given the number of probes to be measured simultaneously.

In conclusion, we have studied the relationship between ERK activity dynamics, Akt activity dynamics, and cell division, and found that simple measures of central tendency of these dynamics in a time coinciding with S/G2 phase are strongly associated with division in single cells. This implies unappreciated roles for ERK and Akt beyond the canonical restriction point. ERK activity is more strongly associated than Akt activity. Yet, neither ERK nor Akt activity perfectly segregate dividing from non-dividing cells, so it is clear other biochemical pathways are important factors for driving single cell division events. ERK and Akt do not interact with one another in the studied contexts, despite the fact that their activities are more correlated in cells that do not divide. These studies conducted in the non-transformed context provide a foundation to explore how cell transformation through oncogenic and/or loss-of-function mutations shape network topology, signaling dynamics, and cell division outcome in cancer, with the potential to identify and target pathway compensation behaviors that promote cell proliferation and survival^94^ in the diseased context. In addition, exploring the role of spatial temporal propagation of ERK and Akt signaling in a 3D tissue context, a model system that MCF10A cells are suited for, can provide insight into how these pathways regulate tissue homeostasis and how transformation disrupts this homeostasis.

## Methods

### Cell culture

MCF10A cells were gifted by Dr. Gordon Mills and cultured in complete sterile filtered (VWR 10040-436) media, consisting of DMEM F12 (Gibco #11330-032) supplemented with 2 mM l-Glutamine (Gibco # 25-005-CI), 20 ng/ml EGF (Peprotech AF-100-15), 10 μg/ml insulin (Sigma #I-1882), 0.5 μg/ml hydrocortisone (Sigma #H-0888), 100 ng/ml cholera toxin (Sigma #C-8052) and 5% horse serum (Invitrogen #16050-122). Cells were passaged with 0.25% trypsin (Gibco #25200056) to maintain sub confluency. Cells were maintained at 37 °C, 5% CO_2_. Starvation media and imaging media is phenol red free DMEM F12 (Fisher #11039-021) supplemented with 100 ng/ml cholera toxin.

HEK293T cells were gifted by the Dr. Dominguez and Dr. Pappapetrou labs and cultured in complete sterile filtered (VWR 10040-436) media, consisting of DMEM (Gibco #11965118) supplemented with 2 mM l-Glutamine (Gibco #25-005-CI) and 10% heat inactivated fetal bovine serum (Gibco #16140071). Cells were passaged with 0.05% trypsin (Gibco #25300054) to maintain sub confluency.

All inhibitors used for KTR validation were formulated as 10 mM stock solutions in DMSO (Sigma Aldrich D2650-5X0ML) and sterile filtered with a 0.22-micron syringe filter. PD0325901 (MEK inhibitor 1) was purchased from Sigma Aldrich (PZ0162-5MG). Trametinib (S2673) (MEK inhibitor 2) and Ipatasertib (S2808) (Akt inhibitor 2) were purchased from Selleck Chemicals. MK2206 (#CT-MK2206) (Akt inhibitor 1) was purchased from Chemietek.

### Imaging

All live cell imaging experiments with a 15 min interval were acquired using an InCell 2200 microscope (GE Healthcare) under environmental control (37 °C, 5% CO_2_) with a Nikon 20X/0.75, Plan Apo, CFI/60 objective. For KTR imaging the following filter sets were utilized: FITC (Excitation: 475/28 nm Emission: 511/23 nm) (ERK-mClover, Akt-mClover KTR); Cy3 (Excitation: 542/27 nm Emission:597/45 nm) (H2B-mRuby2, mCherry-NLS); Cy5 (Excitation: 632/22 nm Emission: 679/34 nm) (ERK-iRFP); Brightfield.

KTR-expressing MCF10A cell lines (pool-see below) were seeded in separate rows of a 96 well plate (Corning #3603) at 5000 cells/well and treated as described. After growth factor and serum starvation, starvation media was aspirated, cells were washed with PBS and 100 μL imaging media was then placed in the wells. Following baseline imaging, cells were treated as indicated by adding 100 μL of 2× solutions in imaging media. Acquired images were processed as described in “Computational image analysis”.

Experiments with a 5-min sampling interval were acquired using the Leica DMI8 (Leica Microsystems) microscope equipped with a HC PL FLUOTAR L 20×/0.40 DRY objective, a pentacube filter (excitation: 458–490 nm, 524–584 nm, 624–754 nm, emission: 500–530 nm, 579–611 nm, 661–701 nm), a Leica DFC 9000GT camera and a CoolLED pE-4000 illumination system. Cells were maintained at 37 °C, 5% CO_2_ using a Pecon environmental control system. 12-bit images were acquired for each probe: Akt-mClover KTR; mCherry-NLS, ERK-iRFP and Brightfield. Raw images were not flatfield corrected or background subtracted due to potential signal loss due to the short acquisition times and exposure settings for this dataset.

### Flow cytometry

EdU flow cytometry assays were performed using the Molecular Probes Click-iT Plus EdU flow cytometry assay kit (C10633 molecular probes). MCF10A cells were seeded in 6 well plates (Corning 353046) at a density of 127 cells/mm^2^ in complete DMEM F12 media. The following day, we serum and serum and growth factor starved cells in DMEM F12 media supplemented with 100 ng/ml cholera toxin for 24 h. Following starvation, cells were pretreated with a final concentration of 100 nM of MEK inhibitor 1 (PD0325901) or 10uM of Akt inhibitor 1 (MK2206) or DMSO control for 30 min. Cells that did not receive inhibitor pretreatment were treated with either MEK inhibitor 1 or Akt inhibitor 1 2, 4, 8, or 12 h post EGF and insulin addition (Final growth factor concentration: 20 ng/ml EGF, 10ug/mL insulin). 22 h post growth factor addition, a final concentration of 10 μM of EdU was added to each well and incubated for 2 h. 2 h post EdU addition, cells were washed with PBS and lifted with 0.25% Trypsin for 10 min. Trypsin was neutralized with complete DMEM F12 media. Cells were pelleted at 100×G for 5 min, resuspended in 100 μl of PBS, and processed as recommended by the manufacturer’s protocol (Molecular Probes Click-iT Plus EdU flow cytometry assay kit). During the last 5 min of permeabilization, 100 μl of diluted 1 μg/ml Hoechst 33342 (ThermoFisher H3570) stain was added. Cells were then washed with a 1% (g/100 ml) bovine serum albumin PBS solution and pelleted at 100 g. Cells were resuspended in permeabilization buffer and stained with EdU Click-iT reaction cocktail for 30 min at room temperature protected from light. Following EdU Click-iT labeling, cells were washed and resuspended in permeabilization buffer and analyzed using the BD Canto II flow cytometer configured with the following laser lines: excitation 640 nm, emission filter 660/20, excitation 405 nm, and emission filter 450/50. Data was gated and processed using FCS Express (Denovo Software).

### Cloning

Akt KTR was modified from the transposase transfection system PSBbi-FoxO1_1R_10A_3D vector (Addgene # 106278)^43^ for lentiviral production (Fig. S6A). The lentiviral expression vector was developed using overlap PCR from fragments generated from the source vector PSBbi-FoxO1_1R_10A_3D: SV40NLS-mCherry-P2A and Gly-FT2DDD-KTR-mClover. Primer sequences are shown in Fig. S6C and were designed in SnapGene and ordered from Sigma Aldrich. Fragments for lentiviral vector construction were generated via PCR using Q5 polymerase (NEB M0491S) and primers specific to SV40NLS-mCherry-P2A and Gly-FT2DDD-KTR-mClover (Fig. S6C) regions of PSBbi-FoxO1_1R_10A_3D (Fig. S6, Fragment 1,2). Fragments were gel purified using the NEB Monarch gel extraction kit (NEB T1020S). Following gel extraction, a 10 cycle PCR reaction was performed using Q5 polymerase and equimolar SV40NLS-mCherry-P2A and Gly-FT2DDD-KTR-mClover fragments using an annealing temperature of 72 °C. 5 μl of the product was amplified using end primers (F: SV40NLS-mCherry-P2A, R: Gly-FT2DDD-KTR-mClover, T_a_ = 69 °C, Fig. S6C). Gateway ATTB sites were inserted at flanking ends using PCR and the ATTB primers (Fig. S6C). The Gateway cloning compatible fragment was inserted into donor vector pDONR221 (Invitrogen™ 12536017) using BP Clonase II (ThermoFisher# 11789020). High Efficiency NEB-5-alpha Competent *E. coli* (NEB C2987I) were transformed with pDONR221 containing the Akt KTR. Transformants were miniprepped with the PureYield Plasmid Miniprep System (Promega A1223) and Sanger sequence verified using GeneWiz. Akt KTR expression vector was generated by performing a LR reaction using pDONR 221-Akt, LR Clonase II (Thermofisher #11791020) and the lentiviral expression vector pLenti CMV Hygro DEST (Addgene #17454) generating the final product, a bi-cistronic hygromycin selectable lentiviral expression vector. The product was transformed into NEB® 5-alpha Competent *E. coli*. Transformations with the correct sequence were maxiprepped with PureYield Plasmid Maxiprep System (Promega #A2392) and used for lentiviral production.

We exchanged the antibiotic selectable marker on the lentiviral expression vector H2B-mRuby2 (Addgene #90236) from hygromycin to puromycin. Specifically, we transferred pLentiPGK Hygro DEST H2B-mRuby2 into pLentiCMV puromycin DEST (Addgene #17452) using BP Clonase II followed by LR Clonase II generating pLentiCMV puromycin DEST H2B-mRuby2 (H2B-mRuby2).

### Lentiviral production

The lentiviral constructs for each cell line are shown in Fig. S6B. Lentiviral particles were generated by transfecting 5 million HEK293T cells seeded in a T75 flask allowed to attach overnight (Corning® T-75 flasks catalog #430641) using the TransIT-293 transfection reagent (Mirus Bio MIR2704) along with expression vector ERK KTR (mClover or iRFP), H2B-mRuby2, or Akt KTR along with packaging plasmid pPAX (Addgene #12260), and envelope protein pCMV-VSV-G (Addgene #8454) according to the manufacturer’s instructions. Two days post transfection, supernatant from was collected and concentrated using Amicon Ultra-15 100 kD centrifugation filters (Millipore #UFC910008). The concentrated lentiviral supernatant was aliquoted and stored at − 80 °C.

### Lentiviral transduction

100,000 MCF10A cells were transduced in suspension in a 6 well plate (Corning 353046) containing complete DMEM F12 medium along with 100 μl lentiviral supernatant. Two days later, expression was validated by fluorescence imaging. ERK KTR MCF10A cell lines (ERK KTR-mClover Hygro, H2B-mRuby2 Puro) were selected in complete DMEM F12 media supplemented with hygromycin (35 μg/ml) and puromycin (2 μg/ml). Akt KTR expressing MCF10A cell lines (SV40nls-mCherry-Akt KTR-mClover Hygro) were selected with DMEM F12 media containing hygromycin (35 μg/ml). Cells were passaged every two to three days in selection media for about 2 weeks. Following selection, cells were expanded in complete DMEM F12 maintenance media containing either both hygromycin (1.5 μg/ml) and puromycin (0.1 μg/ml) (ERK KTR-mClover expressing cells) or hygromycin (1.5 μg/ml) (Akt-KTR-mClover expressing cells). Single clones were not selected, rather, analyses were done with pools. Live-cell imaging was conducted in the absence of selection antibiotics. Dual reporter expressing cells (ERK KTR-iRFP, Akt-KTR-mClover) lines were not selected, as the ERK KTR iRFP (Addgene #59150) lentiviral expression vector does not confer antibiotic resistance. For these, ERK KTR iRFP virus was added to cells for 24 h, and then subcultured as above prior to live-cell imaging analysis.

### Computational image analysis

While many image analysis tools exist^95–97^, each application still requires much novel development tuned to the problem at hand. We developed an automated image analysis pipeline using both iLastik^98^ and CellProfiler^97^ software packages, along with MATLAB scripts (Fig. S2C). It is available at the Birtwistle Lab github repository (github.com/birtwistlelab/Predicting-Individual-Cell-Division-Events-from-Single-Cell-ERK-and-Akt-Dynamics), which includes some dockerized scripts. The analysis pipeline consists of (1) cell nuclei and cytoplasmic segmentation, (2) quantification of KTR fluorescence in both nuclei and cytoplasmic compartments, (3) tracking single cells across a time series, and (4) automatic detection of cell division.

1. Prior to segmentation images were flatfield corrected and background subtracted using CellProfiler. Images of nuclear localized fluorescent protein H2B-mRuby2 (ERK KTR) and NLS-mCherry (Akt KTR) were input into iLastik. Nuclei were identified using a series of features—object intensity, edge detection and texture.
2. The binary mask outputs from iLastik were input into CellProfiler to create a perinuclear ring known as the ‘Cytoring’^65^ which extends 10 pixels from the binary nuclei mask and into the cytoplasm (Fig. S2C). Calculating the cytoplasmic to nuclear KTR fluorescence ratio provides the relative activity of the pathway of interest for that particular cell at that time point.
3. Segmented nuclei identified with iLastik were tracked using CellProfiler’s ***TrackObjects*** module^96,97,99^ and Follow Neighbors^99^. Each identified nucleus was assigned a numerical ID, which corresponds to the same cell across each timepoint. We filter tracks that are shorter than the duration of the time course to prevent quantification of cells that were transiently tracked.
4. Cell division was detected using a feature of cytoplasmic to nuclear KTR fluorescence (C/N ratio) that is unique to dividing cells. As cells divide, there is a change in morphology resulting in a rapid decrease in C/N ratio (Fig. S7). MATLAB’s *findpeaks* function was used to detect when this steep decrease occurs. In dual reporter cells, the Akt KTR was used. We then truncated the time series 5 timepoints before the identified peak, which is attributed to actual kinase activity. For each processed dataset, the output of the cell division script was manually reviewed, and any misclassified cells were removed by directly observing ERK and Akt KTR time course plots for spurious peaks that erroneously resulted in a cell being marked as a dividing cell, or by directly observing the cell nucleus in time-lapse movies for a fusion event. These spurious peaks were more common in the 5-min dataset and could be the result of low dynamic range due to the acquisition parameters. The remaining cells that were not categorized as dividing were defined as non-dividing if the cell was present for 48 h (15-min interval), or greater than or equal to 41.67 h (5-min interval).

The CellProfiler pipeline exports CSV files first preprocessed in Microsoft Excel then analyzed in MATLAB. First, the csv are input into **batchreader.m,** which generates a cell array of tables containing each cell’s measured parameters. The data is then input into the script **ktrTablePlotter.m,** which plots KTR dynamics. Cell division events are detected using the script **Div_detection.m.** For single reporter experiments, ERK or Akt KTR dynamics were utilized for cell division detection. For experiments with cells containing both reporters under 15-min acquisition Akt KTR was utilized for division detection; for 5-min acquisition, ERK KTR was utilized. Division events were manually curated in addition as described above. Cells were separated by division status, and KTR dynamics were plotted for each class.

### Statistics, classification, and visualization

Where box plots are shown, right tailed rank-sum tests were used to calculate p-values for differences between median dividing and non-dividing cells. For logistic regression analysis, individual cell median ERK and Akt activity were calculated across the 8.5–40 h interval. The MATLAB function fitglm, with the following parameters ('Distribution','binomial','link','logit') was applied to cell median activity and cell division response to generate the generalized linear regression model along with its coefficients, and p-values.

NotBoxPlot was retrieved from MATLAB Central File Exchange (Rob Campbell, 2021). Scatter plots of single cell ERK and Akt activity across all timepoints within the 8.5–40 h interval was generated using the MATLAB Central File Exchange script *Scatplot.m*—Alex Sanchez (2020). To assess statistical significance of the correlation coefficient, the mean (µ) and covariance (σ) between ERK and Akt were calculated across all biological replicates and used to sample matched numbers of data points from random multivariate normal distributions for dividing cells and non-dividing cells. This was repeated 1000 times to define the range of correlation coefficients between the 5th and 95th percentiles, which was reported and rounded up to the nearest 0.01.

## Supplementary Information

Supplementary Figures.

## Data Availability

Analysis pipelines can be found on our lab’s GitHub, along with host links for sample datasets (github.com/birtwistlelab/Predicting-Individual-Cell-Division-Events-from-Single-Cell-ERK-and-Akt-Dynamics).
