# Supplementary material for: Relating individual cell division events to single-cell ERK and Akt activity time courses
Source: Sci Rep. 2022 Oct 27;12:18077. doi: 10.1038/s41598-022-23071-6 (PMC9613772; doi:10.1038/s41598-022-23071-6)
Supplement: Supplementary file 1 — Supplementary Figures. [file 41598_2022_23071_MOESM1_ESM.docx]

**Supplementary Figures**

**Figure S1.** **Roles of ERK and Akt activity in cell cycle progression in MCF10A cells.**


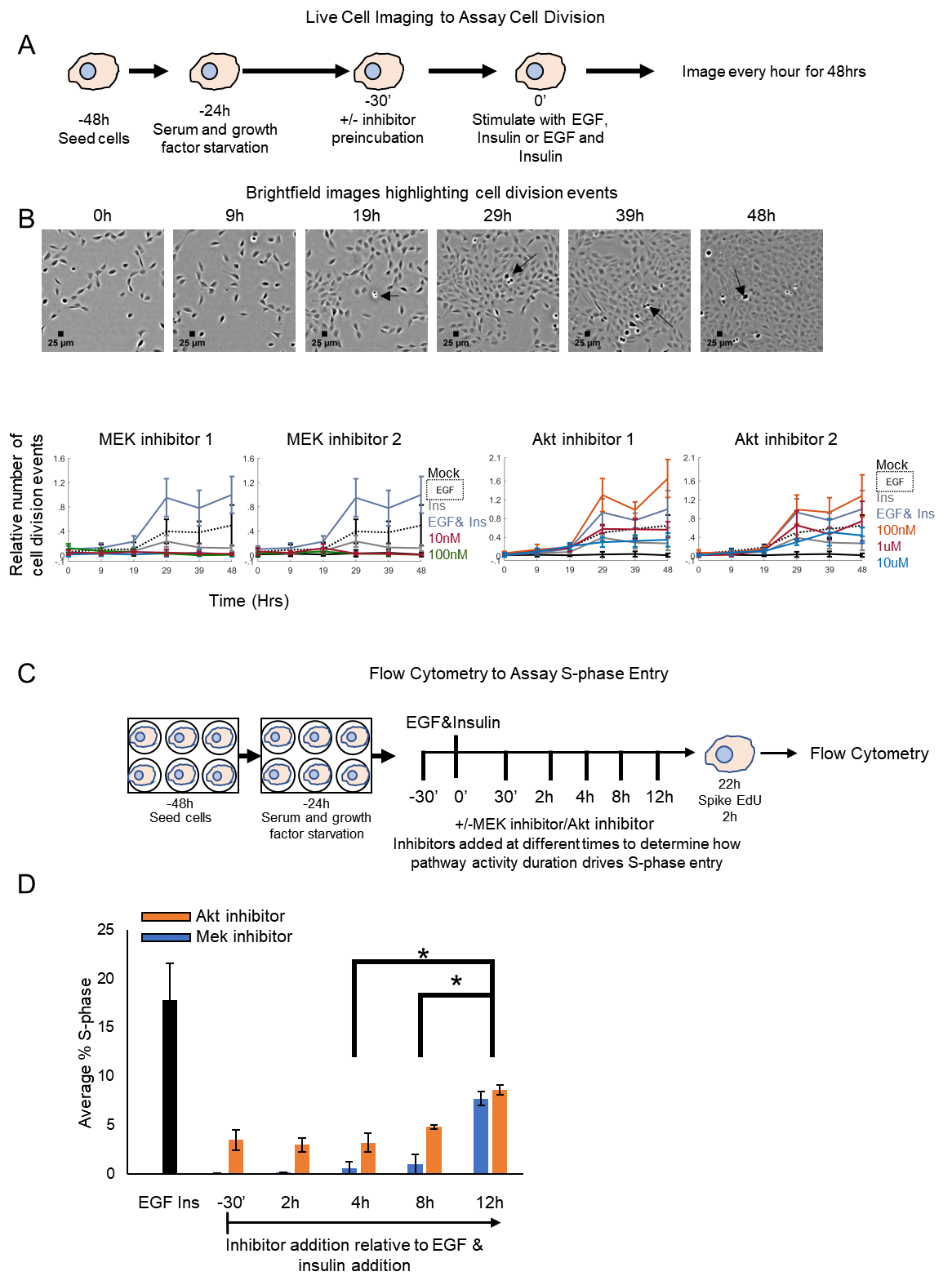


(**A**) Live cell imaging pipeline for quantifying cell division events in MCF10A cells under EGF, insulin, and EGF & insulin stimulation. MCF10A cells were seeded, serum and growth factor starved as described in the methods. Following starvation, cells were pre-incubated with or without MEK inhibitors 1,2 (PD0325901, Trametinib) or Akt inhibitors1,2 (MK2206, Ipatasertib) for 30 minutes. Following inhibitor preincubation, growth factors EGF (20ng/mL), insulin (10ug/mL), or EGF (20ng/mL) & insulin (10ug/mL) were added. Cells were imaged under brightfield every hour for 48 hours. (**B**) Top: Representative brightfield images of cells under EGF and insulin stimulation. Arrows point to representative cell division events. The number of cell division events at selected time points were counted in this manner. Bottom: The relative number of cell division events were calculated by summing the number of observed cell division events across each field of view per condition divided by the total number of observed division events under EGF and insulin stimulation. Error bars represent the standard deviation of normalized cell counts per field per condition. Insulin induces essentially no cell division, but when in combination with EGF, has a more than additive effect. Both ERK and Akt activities appear essential for cell division in this context. (**C**) Experimental outline for relating ERK and Akt dynamics in driving S-phase entry in MCF10A cells. MCF10A cells were seeded, serum and growth factor starved as described in the methods. Post starvation, cells were treated with MEK or Akt inhibitors 1 (PD-10nM and MK-10 uM, according to minimum effective concentrations above) at the times indicated relative to EGF and insulin addition. 22 hours post growth factor addition, EdU was spiked in for 2 hours. (**D**) The average percentage of the cell population in S-phase for each of the conditions shown. A single tailed Student’s t-Test was used to calculate the significance between the average percentage of cells entering S-phase between 4 & 12 hours and 8 & 12 hours at the 95% confidence interval (p< 0.00045). Error bars are from biological triplicates. These results are consistent with the model that time integrated ERK and Akt activities for at least 8-12 hours are necessary for S-phase entry

**Figure S2.** **Live cell imaging assays to establish regimes of validity for kinase translocation reporters**.


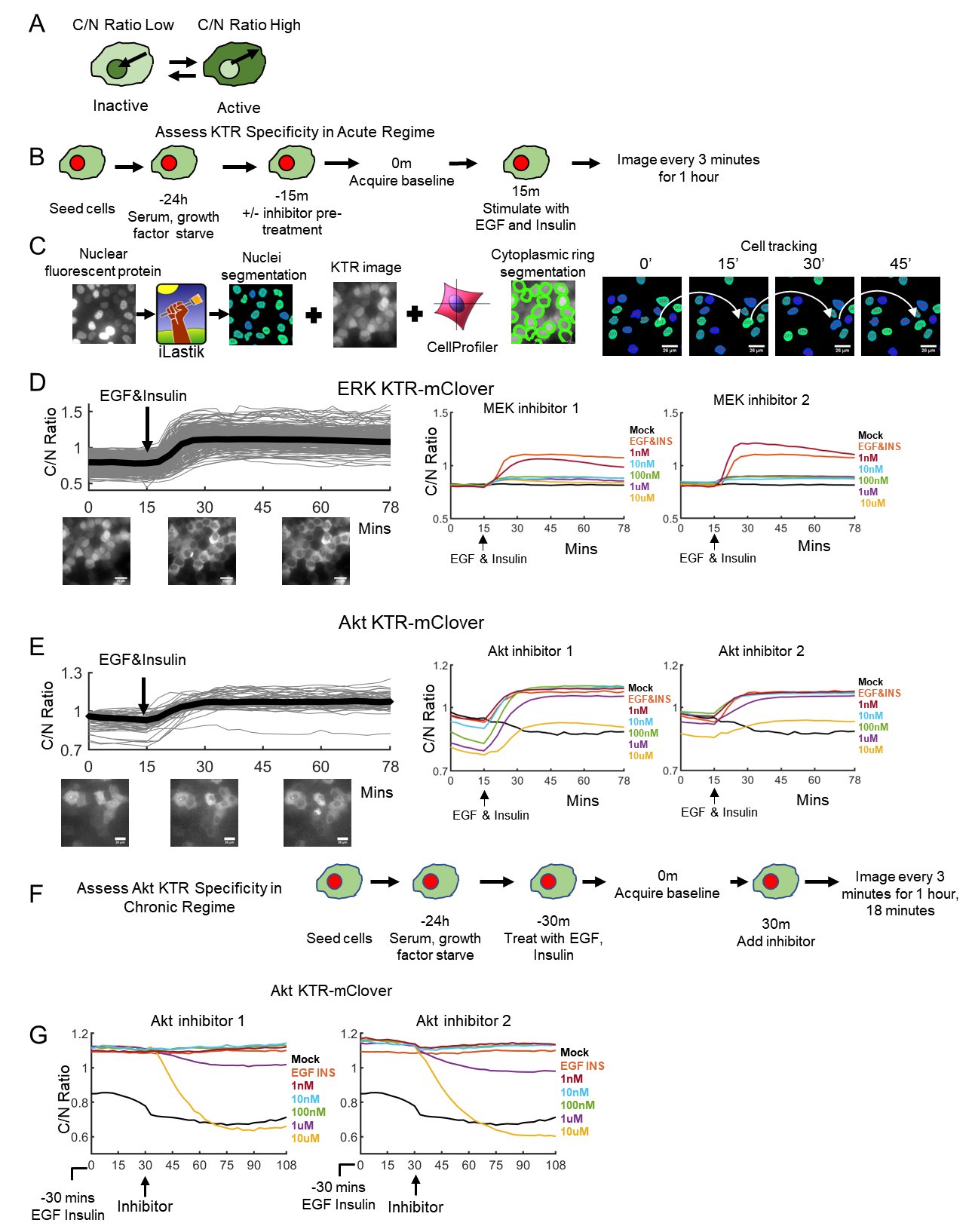


(**A**) Cartoon representation of kinase translocation reporter in the inactive (nuclear) and activated (cytoplasmic) state. (**B**) Acute stimulus kinase translocation reporter validation pipeline. (**C**) Steps in the computational image analysis pipeline for nuclei, cytoplasmic identification, cell tracking and quantification of reported KTR dynamics. (**D**) Single cell traces (gray thin lines) of quantified ERK KTR (C/N Ratio) under EGF (20 ng/mL) and insulin (10 ug/mL) stimulation. Population median (per time point) response is shown in thick black. Representative images of cells expressing ERK KTR are shown below each time point: 0, 30, and 60 minutes. Prior to EGF addition, ERK KTR is mainly nuclear localized (0-15 minutes). By 15 minutes post EGF and insulin addition, ERK KTR is cytoplasmic localized, which is reflected by the increase in the C/N ratio. The panel to the right shows the population median C/N traces for each inhibitor dose condition. KTR activity is ablated with ~10 nM of both MEK inhibitors. MEK inhibitors 1 and 2 are PD0325901 and Trametinib, respectively. (**E**) Single cell traces (gray thin lines) of Akt KTR (C/N Ratio) under EGF (20 ng/mL) and insulin (10 ug/mL) stimulation. Population median (per time point) response is shown in thick black. Prior to EGF and Insulin stimulation, Akt KTR exhibits slightly elevated C/N Ratio, indicative of some basal activity. By 15 minutes post growth factor stimulation, Akt KTR is cytoplasmic localized as shown by C/N Ratio values. Shown to the right are the population median traces for each inhibitor dose condition. Even at 10 uM of each Akt inhibitor, there is significant Akt KTR activity, indicating that at least in the first ~1-hour post-EGF and insulin treatment, the Akt KTR activity likely reports on other kinases. Akt inhibitors 1 and 2 are MK2206 and Ipatasertib, respectively. (**F**) Live cell imaging pipeline to investigate Akt KTR validity under chronic EGF and insulin stimulation. (**G**) Population median Akt KTR dynamics for each inhibitor dose condition. After 1 hour of EGF and insulin treatment, 10 uM of Akt inhibitor completely ablates Akt KTR reporter activity.

**Figure S3. Biological replicates for data presented in Figures 2 and 3.**


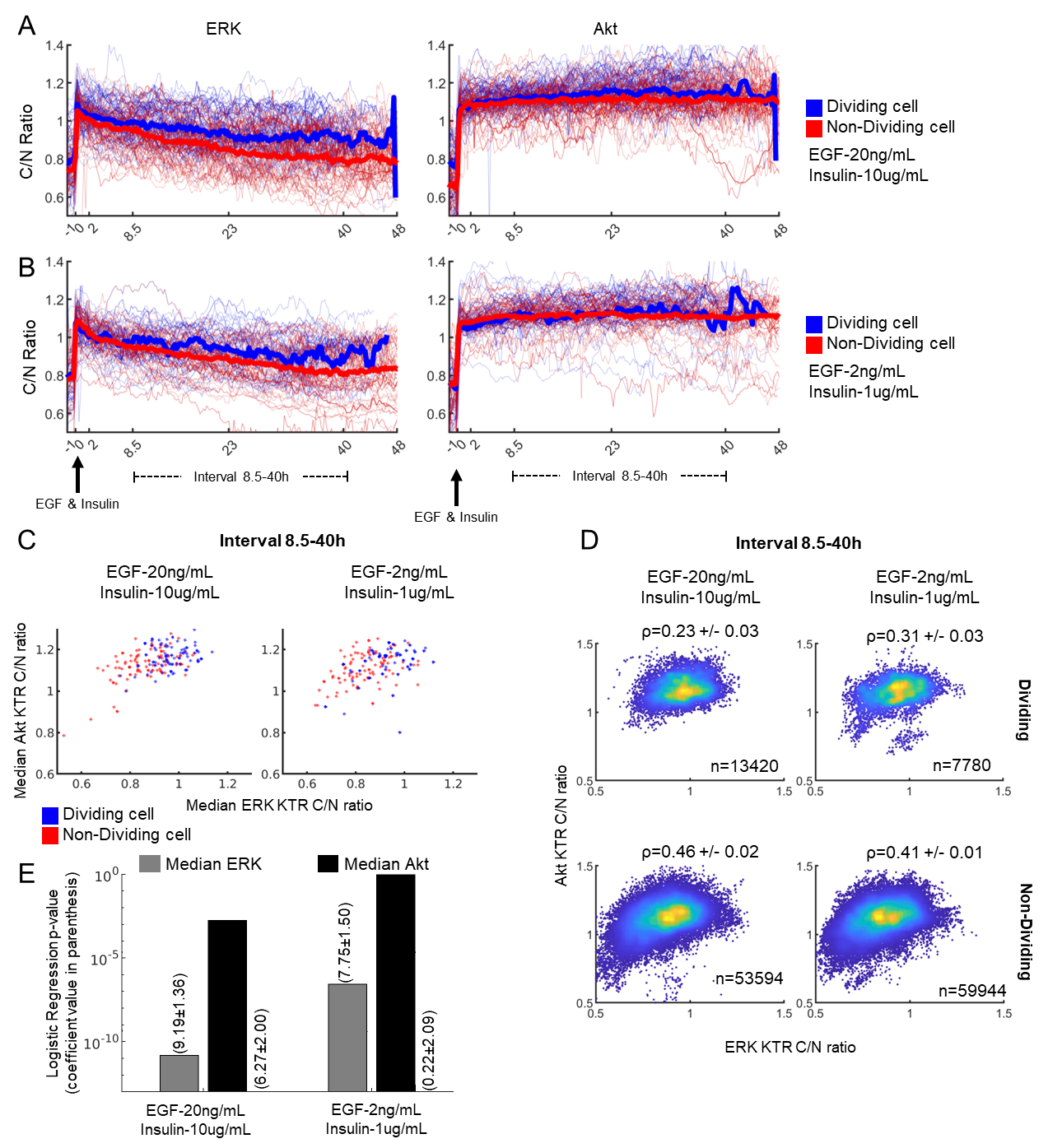


Please see those legends for details

**Figure S4. Biological replicates for KTR range of validity and network reconstruction in the chronic setting using single reporter cell lines.**

MCF10A cells expressing either ERK or Akt KTR were pre-incubated with EGF, and insulin for 90 minutes followed by baseline KTR acquisition. Following baseline, either a MEK or an Akt inhibitor was added. Population median KTR activity calculated across cells for each timepoint are shown for both inhibitors 1 and 2. MEK inhibitors 1,2 are PD0325901 and Trametinib and Akt inhibitors 1 and 2 are MK2206 and Ipatasertib.


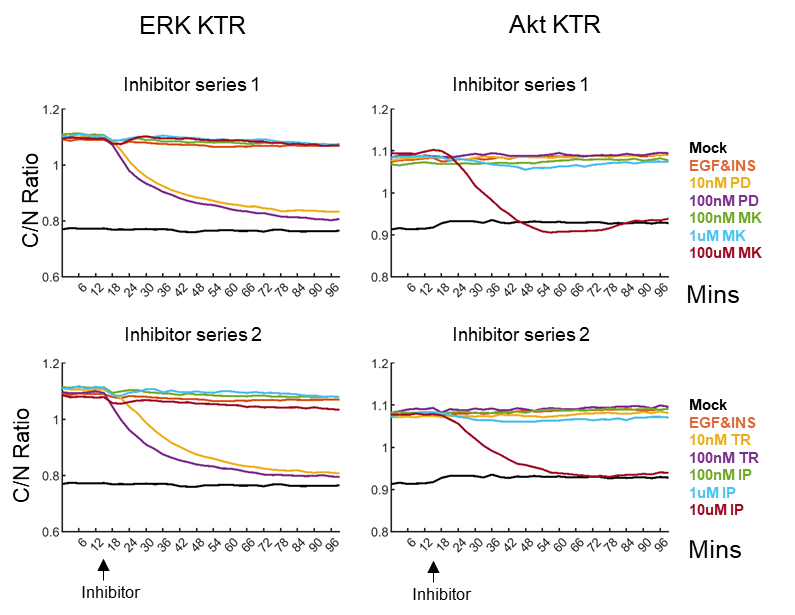


**Figure S5. Biological replicates for chronic regime network reconstruction.**


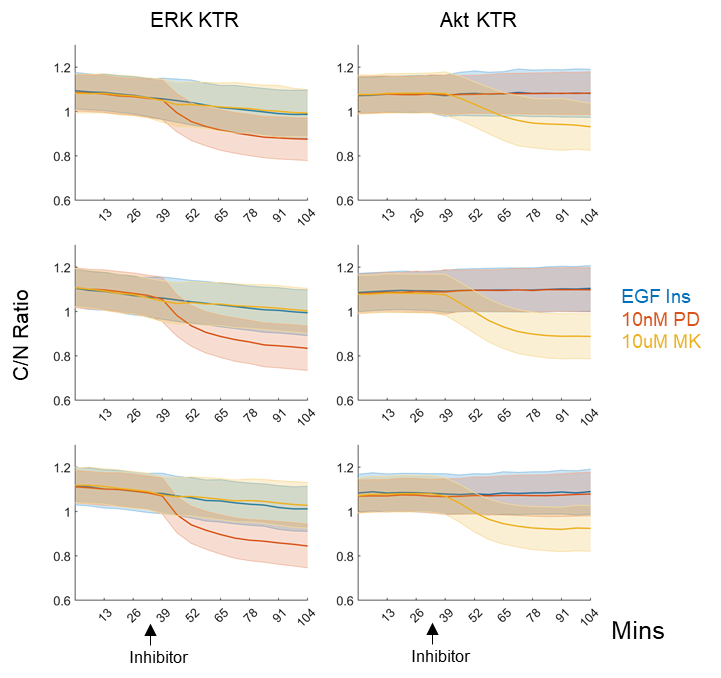


Thick lines are population median (per time point), and shaded regions +/- standard deviations across single cell responses. Please see Figure 3 for more details.

**Figure S6. KTR construction and cell lines.**


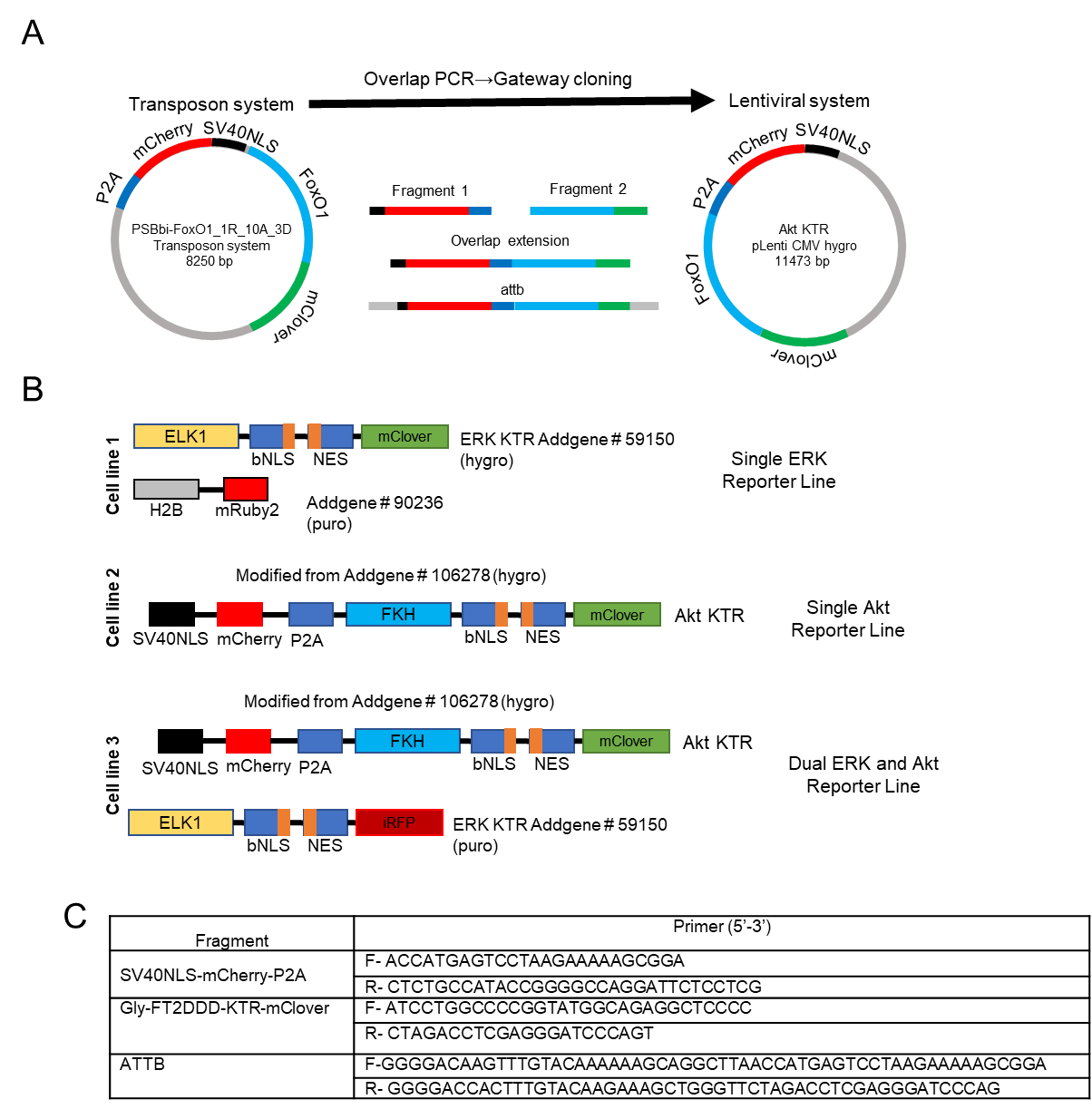


(**A**) Converting the Akt KTR from the transposon backbone to the lentiviral backbone. (**B**) Cartoon representation of constructs used to generate KTR expressing MCF10A cell lines. (**C**) PCR primers used for the different vector construction steps.

**Figure S7. Identification of cell division events.**


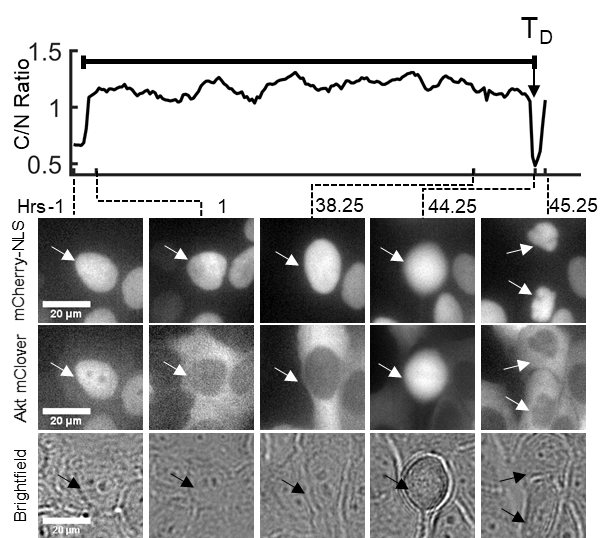


A representative time course and selected images from an Akt KTR expressing MCF10A cell that divides in response to EGF and insulin treatment is highlighted. The nuclear marker is mCherry-NLS. T_D_ denotes the time of division which is determined by the rapid decrease in C/N ratio.

**Figure S8. Association of ERK and Akt Activity Dynamics with Cell Division Fate for 5-Minute Acquisition Times.**


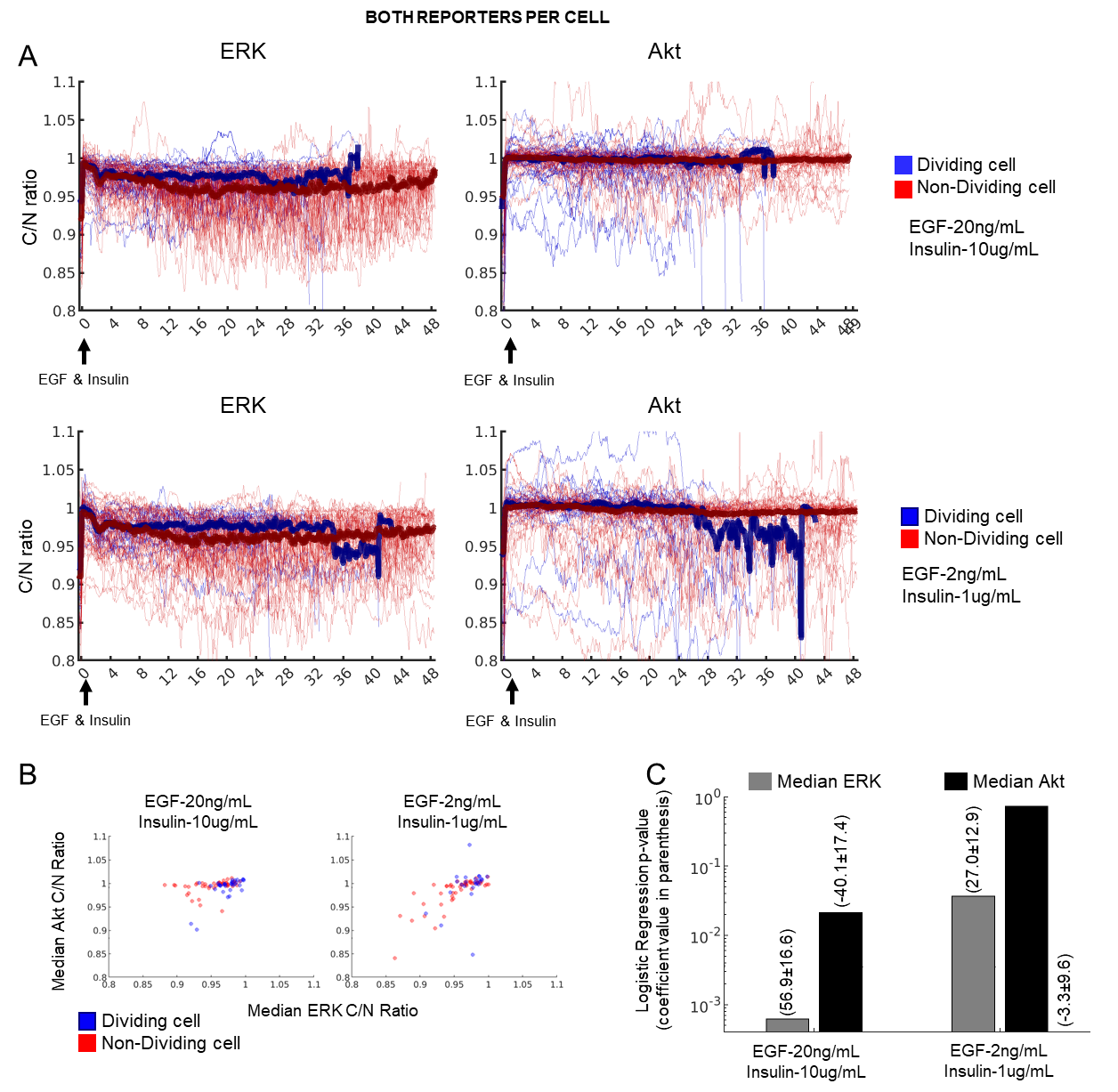


Experimental setup is analogous to that of main text Figure 2, with exceptions noted in Methods

**Figure S9. Selected Cells Showing Dynamic Reporter Range.**


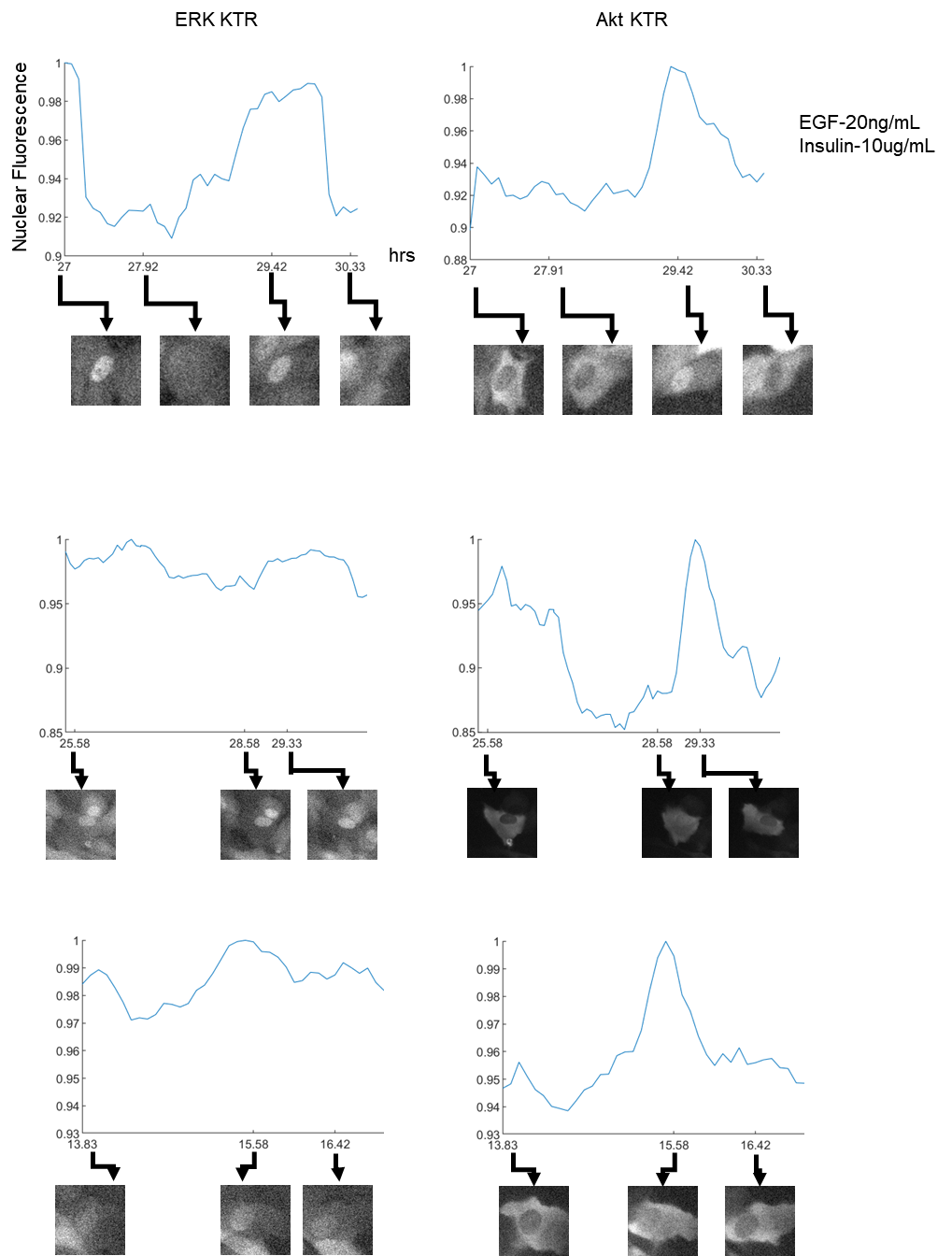


Nuclear fluorescence, as opposed to cytoplasmic / nuclear ratio, is shown on the y-axis. Cell images from different time points are shown to indicate reporter dynamic range at various points in the time courses. They also show differences between ERK and Akt activity in the same single cells. Left is ERK KTR, and right is Akt KTR in the same single cell. The three rows are three different representative cells.

**Figure S10. Cell-to-Cell Variability Over Time.**


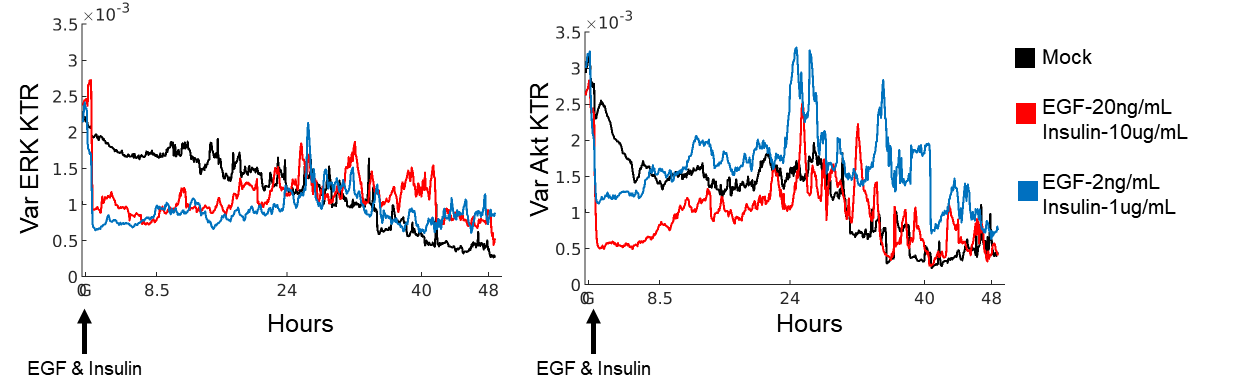


Variance across every quantified cell is plotted vs time to show regimes where the KTR activity is likely to have more dynamic range (higher variance). ERK KTR is on the left and Akt KTR is on the right. Immediately following growth factor treatment, variance decreases sharply and stays low for several hours, indicating a lack of population diversity and potential probe saturation. After a few hours, the variance increases, indicating the opposite.
